# The role of human peritoneal mesothelial cells in the fibrosis and progression of gastric cancer

**DOI:** 10.3892/ijo.2012.1490

**Published:** 2012-05-18

**Authors:** TOMOYA TSUKADA, SACHIO FUSHIDA, SHINICHI HARADA, YASUMICHI YAGI, JUN KINOSHITA, KATSUNOBU OYAMA, HIDEHIRO TAJIMA, HIDETO FUJITA, ITASU NINOMIYA, TAKASHI FUJIMURA, TETSUO OHTA

**Affiliations:** 1 Department of Gastroenterological Surgery, Division of Cancer Medicine, Graduate School of Medical Science, Kanazawa University;; 2 Center for Biomedical Research and Education, School of Medicine, Kanazawa University, Kanazawa, Ishikawa 920-8641, Japan

**Keywords:** gastric cancer, human peritoneal mesothelial cell, fibrosis, epithelial-mesenchymal transition, cell-cell interaction

## Abstract

Peritoneal dissemination is the most frequent metastatic pattern of scirrhous gastric cancer. However, despite extensive research effort, disease outcomes have not improved sufficiently. Tumor progression and metastasis result from interactions between cancer and various cells in the stroma, including endothelial cells, immune cells and fibroblasts. Fibroblasts have been particularly well studied; they are known to change into carcinoma-associated fibroblasts (CAFs) and produce transforming growth factor β (TGF-β), which mediates cancer-stroma interactions. Here, we investigated whether TGF-β derived from cancer cells in the peritoneal microenvironment activates human peritoneal mesothelial cells (HPMCs), leading to the progression and fibrosis of gastric cancer. We found that activated HPMCs (a-HPMCs) took on a spindle shape formation, decreased the expression of E-cadherin and increased that of α-SMA. Furthermore, a-HPMCs became more invasive and upregulated proliferation of human gastric cancer-derived MKN45 cells following direct cell-cell contact. Notably, MKN45 cells co-cultured with a-HPMCs also acquired anchorage-independent cell growth and decreased expression of E-cadherin *in vitro*. To measure the effects of the co-culture *in vivo*, we developed a mouse xenograft model into which different culture products were subcutaneously injected. The largest tumors were observed in mice that had been given MKN45 cells co-cultured with a-HPMCs. Furthermore, these tumors contained HPMC-derived fibrous tissue. Thus, the epithelial-mesenchymal transition (EMT) of HPMCs appears to drive peritoneal dissemination and tumor fibrosis.

## Introduction

The diffusely infiltrating human scirrhous gastric carcinoma is characterized by cancer cell infiltration and proliferation accompanied by extensive stromal fibrosis. The most frequent metastatic pattern of scirrhous gastric carcinoma is peritoneal dissemination, for which anticancer therapies have been developed. However, despite the proven benefits of these treatments (1,2), outcomes of this carcinoma require further improvements.

Although cancer research has traditionally focused on malignant cancer cells, recent developments have shown that tumor progression is the product of interactions between cancer cells and various cells in the stroma, such as endothelial cells, immune cells, and fibroblasts (3–6). Fibroblasts have been particularly well-studied (7); for instance, the presence of orthotopic fibroblasts has been shown to contribute to cell growth and extensive stromal fibrosis at the primary cancer site (8). Furthermore, it is known that growth factors derived from orthotopic fibroblasts are not adequate for enhancing gastric cancer invasiveness; a direct interaction between cancer cells and orthotopic fibroblasts is important for acceleration of cancer cell invasion and progression (9). At the peritoneal dissemination site, cancer cells usually generate a supportive microenvironment by producing stroma-modulating growth factors (fibroblast growth factor [FGF] family, platelet-derived growth factor [PDGF], epidermal growth factor [EGF] ligands, vascular endothelial growth factor [VEGF] family, interleukins, and transforming growth factor-β1 [TGF-β1]) (10,11). These factors activate fibroblasts and facilitate additional secretion of growth factors and proteases. As a result, scirrhous gastric cancer cells stimulate the proliferation of peritoneal fibroblasts and induce peritoneal fibrosis.

Fibroblasts within the cancer stroma are known as carcinoma-associated fibroblasts (CAF), and acquire a modified phenotype (12,13). Cancer cells are modified by CAFs and induce epithelial-mesenchymal transition (EMT)-like changes characterized by repression of E-cadherin expression and enhancement of α-SMA expression. Generally, cell transformation progresses gradually, rather than drastically in the EMT process. However, under these cell-cell interactions, each cell is facilitated by a characteristic change into irreversible transformation (14).

Fibroblasts are the principal effectors mediating fibrosis. In the tumor microenvironment, activated fibroblasts arise from several sources, such as resident fibroblasts or myofibroblasts (15), bone marrow-derived cells or fibrocytes (3,16), and both endothelial- and epithelial-mesenchymal transitions (EndMT and EMT, respectively) (5).

In scirrhous gastric cancer, fibrous tissue volume and composition are regulated by the response of fibroblasts to the growth factors that are released by cancer cells (17,18). Increased expression of TGF-β1, in particular, has been demonstrated in fibrotic tissue and regions of increased extra-cellular matrix deposition in various organs (19,20). TGF-β1 is also known as the crucial inducer of EMTs, which play an important role in the development and malignancy of various human carcinomas (21). Loss of epithelial character is typically observed late in the progression of human carcinomas, and correlates with their growth, local invasion, angiogenesis, and metastasis (22).

Human peritoneal mesothelial cells (HPMCs), which are classified as epithelium in the broad sense of the term, serve as a protective anatomical barrier and play a key role in the immunological response to infection, wound healing, and the dissemination of tumor cells such as those responsible for ovarian cancers (23). HPMCs also produce EGF, FGF and VEGF, and their expression is significantly upregulated in the peritoneal cavity following surgical intervention, when free cancer cells may be spilled during the course of the resection (24). In cancerous environments, HPMCs frequently undergo a change in morphology, taking on a fibroblastic shape rather than their usual epithelial-like formation - a change that, along with fibrosis of the peritoneum, was recently linked to TGF-β1 activity (25,26). These physical alterations expose submesothelial connective tissue and facilitate adhesion of cancer cells (27). Activated HPMCs also acquire migratory potential (25), but their proliferation potential and their possible interactions with cancer cells remains unknown.

In this study, we investigated whether activated HPMCs (a-HPMC) induced by TGF-β1 stimulation contribute to gastric cancer cell infiltration, proliferation, and fibrosis. We documented the behavior of HPMC, a-HPMC, and gastric cancer cells in the presence or absence of direct co-culture with HPMCs. We also used a mouse xenograft model to study the interactions of gastric cancer cells and HPMCs or a-HPMCs. We found that HPMCs exposed to TGF-β1 acquire a fibroblast-like formation and the ability to invade. Furthermore, presence of a-HPMCs contributes to cancer cell growth and fibrosis under conditions of direct cell contact in the tumor microenvironment.

## Materials and methods

### Cell lines and cell culture

HPMCs were isolated from surgical specimens of human omentum as previously described (28). Omental specimens were obtained, with informed consent, from patients undergoing elective abdominal surgery. Tissue samples were collected in ice-cold phosphate buffered saline (PBS) to minimize cell degeneration. Samples were immediately washed extensively in PBS to remove contaminating red blood cells and incubated with pre-warmed PBS containing 0.125% trypsin/EDTA (Gibco, USA) for 30 min at 37°C. The suspension was passed through a 100-*μ*m-pore nylon mesh (Becton Dickinson, Japan) to remove undigested fragments, then centrifuged at 1,500 rpm for 5 min. The collected cells were cultured in RPMI 1640 medium (Gibco) supplemented with 10% heat-inactivated fetal bovine serum (FBS; Nichirei Bioscience Inc., Japan), 100 IU/ml penicillin, 100 mg/ml streptomycin (Gibco), and 2 mM glutamine (Nissui Pharmaceutical Co., Ltd., Japan). Cells were seeded in a gelatin-coated 75-cm^2^ dish flask (BD Falcon, USA) and cultured in 10 ml of medium at 37°C in a humidified atmosphere of 5% CO_2_ in air.

Human gastric cancer cell lines (MKN45) were obtained from the American Type Culture Collection (Rockville, USA). The culture medium for MKN45 cells was RPMI-1640 (Gibco) with the same additives as mentioned above. MKN45 cells were grown to confluence and harvested by trypsinization with 0.25% trypsin/EDTA. The confluent HPMCs were trypsinized with 0.125% trypsin/EDTA prior to use. HPMCs were then transferred to serum-free medium for 24 h, after which they were continuously exposed to 5 ng/ml of recombinant human TGF-β1 (Sigma-Aldrich, Inc., USA) for 48 h. Finally, they were transferred to RPMI-1640 containing 10% FBS, which caused the cells to undergo a shift in morphology, allowing us to identify the resulting cells as activated HPMCs (a-HPMCs). HPMCs were used at passage 1–3 in all experiments.

### Invasion assay

The effects of TGF-β1 treatment on cell invasion were determined using a BD BioCoat Matrigel Invasion Chamber for 24-well plates (BD Bioscience, USA), according to the manufacturer’s instructions. First, Matrigels were rehydrated using 750 *μ*l of serum-free medium, after which we added 750 *μ*l of fresh medium containing 10% FBS to the lower chamber. Next, 0.5 ml of HPMC and a-HPMC cells (1×10^5^ cells/ml) in serum-free media were seeded into the upper chamber of the system (both the control membrane and Matrigel membrane were seeded with cells). After 24 h of incubation, the cells in the upper chamber were removed and the cells that had invaded through the Matrigel membrane were stained by hematoxylin and fixed in 100% methanol. Membranes were removed from inserts and mounted on slides. Invading cells were counted using a microscope with a 10× objective in several fields of triplicate membranes. Data are expressed as the percentage of invasion through the Matrigel membrane relative to the migration through the control membrane.

### Cell proliferation assay

MKN45 cells, seeded at a density of 1×10^5^ cells per well in 6-well plates, were incubated alone (control) or in the presence of a direct co-culture with the same number of HPMCs or a-HPMCs. A 1-*μ*m pore Boyden Chamber (BD Falcon) was used for indirect incubation. Cells were counted on Days 1, 3, and 5 post-seeding. The magnetic-activated cell sorting (MACS, Miltenyi Biotec, Germany) method was used to separate MKN45 cells from HPMCs and a-HPMCs. Following trypsinization, microbead-labeled anti-human CD326 antibody (Miltenyi Biotec) was used to label cells. Data were averaged across each experiment (for example, ‘with co-culture’ and ‘without co-culture’), for which sample points were assayed in triplicate and results were counted twice.

### Transformation assay

Soft agar assays were performed in 6-well plates. The assay medium comprised equal volumes of 2-fold concentration RPMI-1640 containing 20% FBS and 1.44% agar solution (final concentration = 0.72%). Individual wells were coated with 1.5 ml of base medium.

The cells (HPMC, a-HPMC, MKN45, MKN45 co-cultured with HPMC, MKN45 co-cultured with a-HPMC) were harvested with trypsin, resuspended in RPMI-1640 containing 10% FBS, and mixed with an equal volume of the aforementioned assay medium (final concentration = 0.36%). Co-culture cells were incubated for 5 days and isolated by MACS. In all soft agar cultures, cells were seeded on solidified base layers in a 2-ml volume at a density of 1×10^4^ cells/ml. These cultures were incubated for 14 days and counted using a microscope with a 4× objective in several fields. Colonies containing >5 cells were scored as positive. All examinations were performed in triplicate.

### Western blotting

Cells were lysed in RIPA buffer (50 mmol/l Tris-HCl (pH 8.0), 150 mmol/l sodium chloride, 0.5 w/v% sodium deoxycholate, 0.1 w/v% sodium dodecyl sulfate, 1.0 w/v% NP-40 substitute) (Wako, Japan) containing 1% protease inhibitor cocktail (Sigma-Aldrich Inc.). The protein concentration of each sample was measured using a BCA protein assay kit (Pierce Biotechnology, USA). Whole-cell lysates were prepared in denaturing SDS sample buffer and subjected to SDS-PAGE (ATTO Co. Ltd., Japan). The immunoblots were visualized using an ECL Plus kit (GE Healthcare UK Ltd.). We employed the following primary antibodies: E-cadherin (H-108, rabbit polyclonal IgG, diluted 1:1,000; Santa Cruz Biochemistry, USA), α-SMA (1A4, mouse monoclonal IgG, diluted 1:5,000; DakoCytomation, Denmark), and β-actin (AC-15, mouse monoclonal IgG, diluted 1:10,000; Sigma).

### Mouse xenograft model

All animal experiments were performed according to Kanazawa University’s standard guidelines. We used BALB/c nu/nu mice (female, 4–6 weeks old; Charles River Laboratories Inc. Japan) as a xenograft model in which we investigated the effects of co-culturing MKN45 with HPMCs and a-HPMCs. HPMCs and a-HPMCs were first stained with a red fluorescent dye PKH26 cell linker kit (Sigma) according to the manufacturer’s instructions; the concentration of PKH26 during incubation was 4 *μ*M. Next, MKN45 cells were co-cultured with an equivalent number of HPMCs or a-HPMCs for 5 days. A total of 5×10^6^ cells in 100 *μ*l of RPMI-1640 were then subcutaneously injected into the dorsal side of each mouse (n=27). The mice were divided into the following experimental groups: a control group (MKN45 s.c., n=9), MKN45 co-cultured with HPMC group (n=9), and MKN45 co-cultured with a-HPMC group (n=9). Xenograft tumors were measured every other day for 10 days. Tumor volume was estimated using the equation *v=(ab^2^)/2*, where *v* is volume, *a* is the length of the major axis, and *b* is the length of the minor axis. At the end of the experiment, tumor specimens were collected for immunohistochemical examination.

### Histological and immunohistochemical examination

Tumor specimens obtained from subcutaneous tumors were shock frozen in liquid nitrogen for fluorescence microscopy. Specimens were cryosectioned and mounted on a glass slide, air dried, and immediately analyzed by fluorescence microscopy using a standard filter setup for visualization of PKH26. Tumor specimens were then fixed in 10% neutral buffered formalin and embedded in paraffin. Sections were stained with hematoxylin and eosin (H&E) and Azan stain, and immunostained with E-cadherin antibody (H-108, rabbit polyclonal IgG, diluted 1:100; Santa Cruz Biochemistry) and α-SMA (1A4, mouse monoclonal IgG, diluted 1:100; DakoCytomation) at 4°C overnight. The sections were treated with EnVision reagent (Dako Co., Japan) for visualization.

### Statistical analysis

We investigated differences among the data sets by one-way analysis of variance (ANOVA) or two-sided Student’s t-tests using the computer software package SPSS 10.0 (SPSS Inc., USA). P<0.05 indicated a statistically significant difference.

## Results

### Effect of TGF-β1 on the invasiveness and anchorage-independent growth of human peritoneal mesothelial cells in vitro

**Figure 1:**
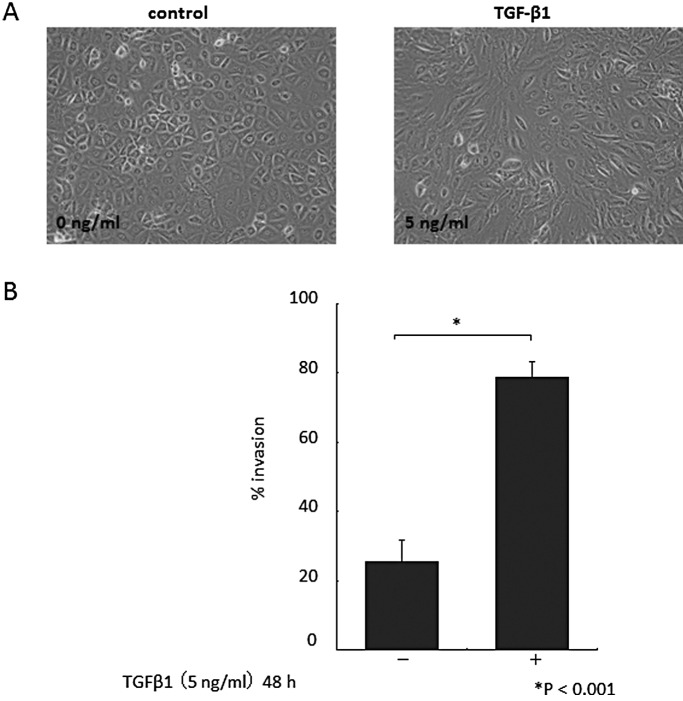
(A) Representative image of morphological changes induced 48 h after the introduction of TGF-β1 to HPMC cultures. HPMCs cultured in control medium (left) or in medium containing 5 ng/ml of TGF-β1 (right) were visualized by phase contrast microscopy at magnification, ×200. (B) Effect of TGF-β1 on the invasiveness of HPMCs *in vitro*. The experiment was conducted 3 times and each sample was assessed in triplicate; data are expressed as the means ± SE.

**Figure 2:**
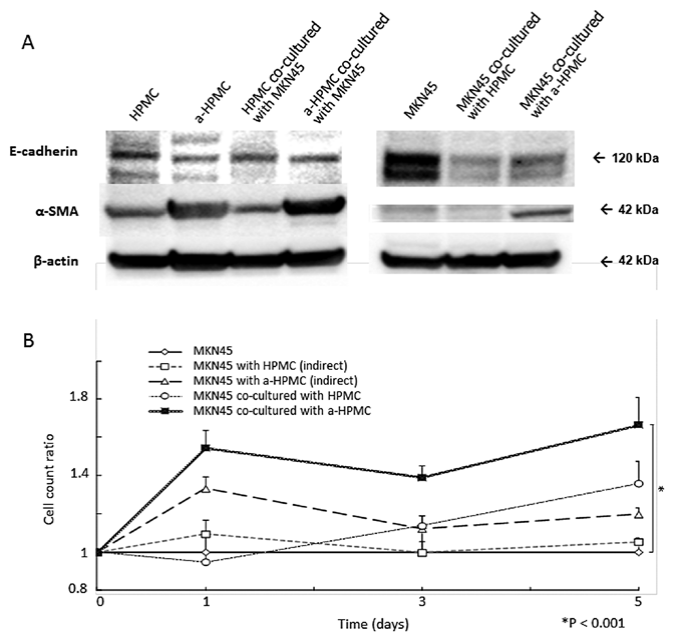
(A) Results of the western blot analysis assaying for E-cadherin (120 kDa) and α-SMA (42 kDa). Expression of α-SMA was higher in a-HPMCs than in HPMCs, while the reverse was true for the expression of E-cadherin. The effect of direct co-culture was unclear. E-cadherin expression was markedly lower in MKN45 cells co-cultured with HPMCs than in MKN45 cells alone. It is not clear whether the high level of α-SMA expression in the MKN45 cells co-cultured with a-HPMC was a genuine effect or a result of contamination by a-HPMC. (B) Effect of directly and indirectly co-culturing MKN45 cells with HPMCs (HPMC, a-HPMC). MKN45 cells were counted on Days 1, 3, and 5 post-seeding. Cell proliferation was evaluated by comparing observed cell numbers to those found in normal (MKN45-only) cultures. The experiment was conducted 3 times and each sample was assessed in triplicate; data are expressed as the means ± SE.

Morphological changes in cultured HPMCs were observed 48 h after the addition of TGF-β1. HPMCs cultured without TGF-β1 had a cobblestone-like appearance (Fig. 1A, left). By contrast, HPMCs treated with TGF-β1 displayed a spindle fibroblastic pattern morphology (Fig. 1A, right). These changes are typical of cells with a mesenchymal phenotype. TGF-β1 exposure increased the invasiveness of HPMCs (P<0.001; Fig. 1B). As expected, given the morphological changes, western blot analysis showed a decrease in E-cadherin expression and an increase in α-SMA expression (Fig. 2A).

**Figure 3:**
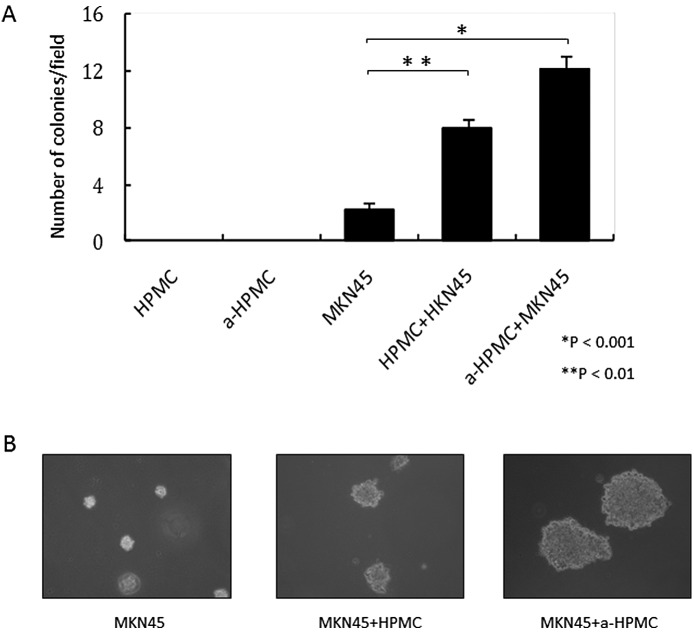
Results from the transformation assay investigating anchorage-independent cell growth. (A) Number of colonies per cell line. Data were averaged across 3 repeats of each experiment, in which each sample was counted across 6 fields (×100). (B) Colony formation of MKN45 cells, MKN45 cells co-cultured with HPMC, and MKN45 cells co-cultured with a-HPMC. Images were captured on the 14th day of culture using a phase-contrast microscope (original magnification, ×100).

HPMCs were not able to grow in soft agar gel, regardless of whether or not TGF-β1 was present (Fig. 3A and B). Thus, treatment with TGF-β1 was responsible for the acquired abilities of mobility and invasiveness through the basal membrane. However, HPMCs did not independently acquire anchorage-independent growth.

### Effect of a-HPMC co-culture on the proliferation of gastric cancer cells

MKN45 cell count was elevated after co-culturing with a-HPMCs (P<0.001; Fig. 2B). However, when MKN45 and a-HPMCs were separately cultured using Boyden Chamber inserts, no significant increase in MKN45 cells was detected (Fig. 2B). In addition, MKN45 cells co-cultured with a-HPMCs were able to form larger and greater colonies in soft agar gel (Fig. 3A and B).

No morphological changes of MKN-45 cells during this assay were observed (data not shown). However, our results showed that cell-cell contact with HPMCs attenuates intra-cellular expression of E-cadherin (Fig. 2A) in MKN45 cells. Elevation of α-SMA expression was also observed in MKN45 cells co-cultured with a-HPMCs. This result indicates that the MACS method was not able to eliminate cell contamination.

### Effects of a-HPMCs in subcutaneous xenograft models

**Figure 4:**
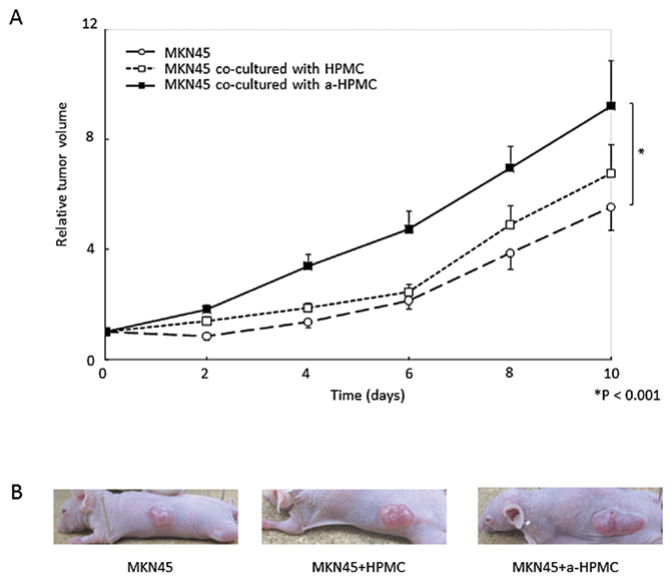
(A) Results from the *in vivo* subcutaneous xenograft model investigating the effects of co-culturing with HPMCs. Tumor volumes were measured every other day and calculated as the product of the length, width, and height of each tumor. Results are expressed as the means ± SE (n=9). (B) Representative images showing the macroscopic appearance of the tumors at Day 9.

**Figure 5:**
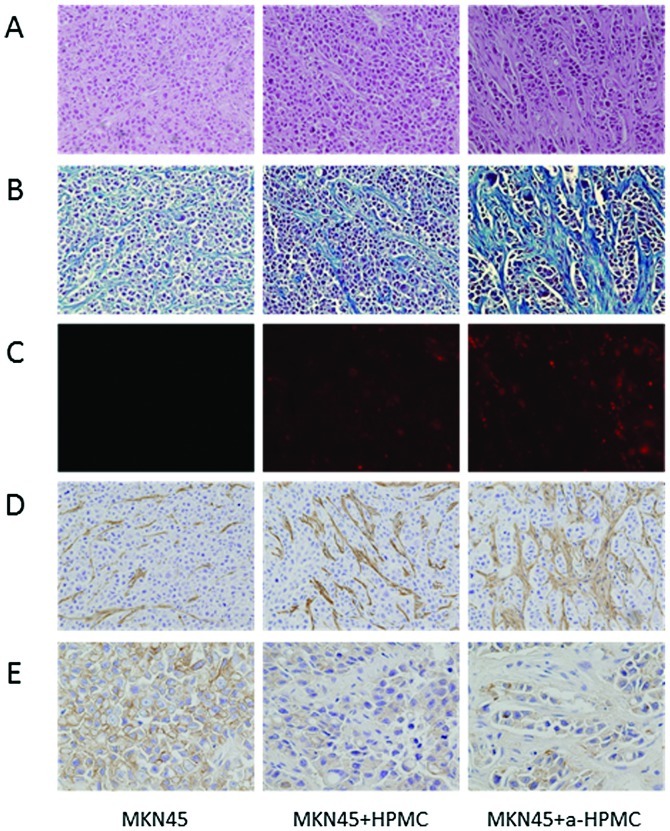
Microscopic views of mouse xenograft tumors. (A) Histological examination was performed by hematoxylin and eosin (H&E) staining. (B) Fibrotic tissue as determined by AZAN staining in the subcutaneous xenograft tumors 10 days after inoculation. (C) Results from fluorescence microscopy (original magnification, ×200) investigating implantation of HPMC and a-HPMC in subcutaneous xenograft tumors. Red indicates labeled HPMCs. Immunohistochemical examination of (D) αSMA and (E) E-cadherin in subcutaneous xenograft tumors (original magnifications, ×200 and ×400, respectively).

The time course of subcutaneous relative tumor volume is shown in Fig. 4A. At 10 days post-inoculation, the mean relative volume of tumors created with MKN45 cells co-cultured with a-HPMCs was significantly larger than that of the other groups (P<0.001). However, we did not observe any morphological differences in the subcutaneous tumors (for example, scirrhous or medullary types) (Fig. 4B). The area of fibrosis was significantly larger in tumors created from MKN45 cells co-cultured with a-HPMCs than in tumors from the other groups (Fig. 5B). We confirmed implantation of the subcutaneous tumors and HPMCs labeled by PKH26 cell linker kit (Fig. 5C); in tumors from MKN45 cells co-cultured with a-HPMCs, there were higher numbers of implanted cells and higher levels of fibrosis.

Expression of α-SMA in the subcutaneous tumors was highest in animals implanted with MKN45 cells co-cultured with a-HPMCs, which corresponds to the notable increase in fibrous tissue (Fig. 5D). Expression of E-cadherin was lower in MKN45 cells co-cultured with both HPMCs and a-HPMCs than in MKN45 cells cultured alone. This suggests that HPMCs were responsible for the reduction in E-cadherin expression (Fig. 5E).

## Discussion

**Figure 6:**
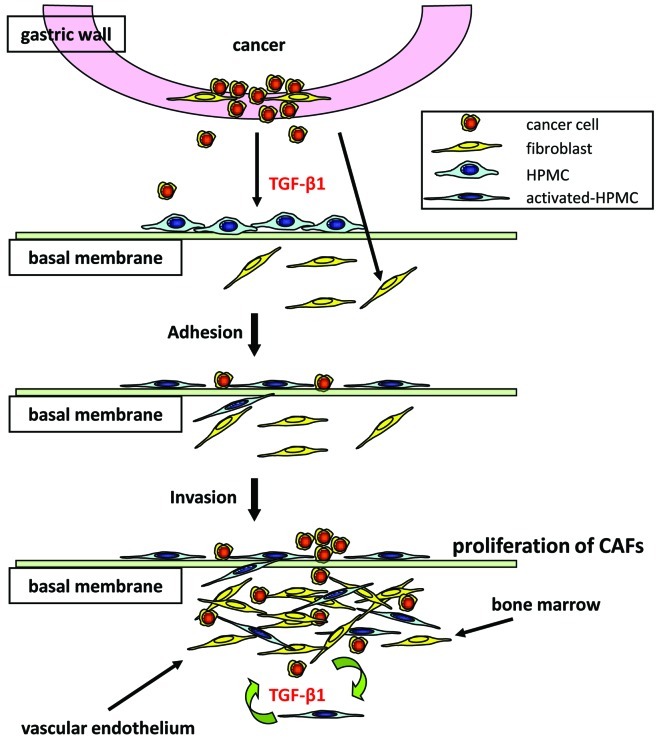
Scheme illustrating the potential activity of HPMC at peritoneal metastatic sites. First, TGF-β1 activates HPMC; next, activated HPMC infiltrates the basal membrane and induces EMT of gastric cancer. This contributes to tissue fibrosis as well as CAFs by direct contact with gastric cancer cells.

We have shown that tumor fibrosis is produced not only by orthotopic fibroblasts, but also by HPMCs undergoing mesenchymal transition; furthermore, we have demonstrated that direct physical contact with cancer cells drives the process of fibrosis (Fig. 6). HPMCs stimulated by TGF-β1 undergo morphological changes (Fig. 1A) and also acquire the ability to invade the basal membrane *in vitro* (Fig. 1B). We found that direct contact between gastric cancer cells and a-HPMCs contributes to the growth and transformation of gastric cancer cells (Figs. 2 and 3). As a result, activity of a-HPMCs on the basal membrane causes highly fibrotic changes *in vivo* (Figs. 4 and 5).

Compelling evidence suggests that morphological changes in HPMCs are caused by TGF-β1 derived from gastric cancer cells and host fibroblasts (29,30); expression of this protein causes the exposure of submesothelial connective tissue and adhesion of cancer cells. In our current mouse xenograft model, a-HPMCs implanted in greater quantities, and induced more fibrosis than HPMCs (Figs. 5A and B). a-HPMCs acquire invasiveness *in vitro* and contribute to cancer cell proliferation as CAFs do (Figs. 1B and 2). CAFs are derived from various cells, including local fibroblasts, bone marrow fibrocytes, and endothelial cells (3,5,16). Our study is the first to suggest that a-HPMCs are also a possible source of CAFs.

We found that direct contact with a-HPMCs led to anchorage-independent growth (Fig. 3) and repression of E-cadherin expression (Figs. 2 and 5) in gastric cancer cells. This indicates that a-HPMCs contribute to the EMT-like change observed in gastric cancer cells following cell-cell contact. However, cancer cells co-cultured with normal HPMCs also have reduced E-cadherin expression (Fig. 2). According to recent reports, CAFs are implicated in cancer by producing high quantities of stromal-derived factor-1α (SDF-1α), also known as chemokine CXCL12 (31); SDF-1α expression is higher in mesothelial cells than in other organs (32). Although we hypothesized that differences in SDF-1α expression may be responsible for the differential activity of HPMCs and a-HPMCs observed in our study, we found no evidence to support this (data not shown). Thus, future research should attempt to elucidate the mechanism behind the different effects of HPMCs and a-HPMCs on gastric cancer cells.

In conclusion, we have shown that HPMCs activated by TGF-β1 signaling acquire their own invasiveness and promote fibrosis in a mouse xenograft model. In addition, direct contact with a-HPMCs contributes to EMT-like changes in gastric cancer cells and promotes tumor growth. Our results strongly suggest that HPMCs are one of the origins of CAFs; therefore, understanding the mechanism of EMT in HPMCs is necessary for developing a molecular approach to combat peritoneal dissemination of gastric cancer.
